# Relationship Among Physical Activity Level, Mood and Anxiety States and Quality of Life in Physical Education Students

**DOI:** 10.2174/1745017901713010082

**Published:** 2017-08-09

**Authors:** Sandro Legey, Filipe Aquino, Murilo Khede Lamego, Flavia Paes, Antônio Egídio Nardi, Geraldo Maranhão Neto, Gioia Mura, Federica Sancassiani, Nuno Rocha, Eric Murillo-Rodriguez, Sergio Machado

**Affiliations:** 1 Laboratory of Panic and Respiration, Institute of Psychiatry of Federal University of Rio de Janeiro (IPUB/UFRJ), Rio de Janeiro, RJ, Brazil; 2 Multidisciplinary Laboratory of Physical Activities, Sports and Physical Education (LAMAFEEF/UVA), Veiga de Almeida University, Cabo Frio, RJ, Brazil; 3 Physical Activity Sciences Postgraduate Program - Salgado de Oliveira University (UNIVERSO) Niterói, Brazil; 4 Department of Public Health, Clinical and Molecular Medicine, University of Cagliari Cagliari, Italy; 5 School of Allied Health Sciences, Polytechnic Institute of Porto, Porto, Portugal; 6 Laboratorio de Neurociencias Moleculares e Integrativas, Escuela de Medicina, División Ciencias de la Salud, Universidad Anáhuac Mayab. Mérida, Yucatán, México; Grupo de Investigación en Envejecimiento. División Ciencias de la Salud, Universidad Anáhuac Mayab. Mérida, Yucatán. México; 7 Physical Activity Neuroscience Laboratory, Physical Activity Sciences Postgraduate Program - Salgado de Oliveira University (UNIVERSO), Niterói, Brazil; 8 Intercontinental Neuroscience Research Group

**Keywords:** Physical activity level, Leisure-time Physical activity, Anxiety, Mood state, Quality of life, Mental health

## Abstract

**Background:**

Physical activity level (PAL) is known to play an important role in reducing risk factors associated with sedentarism, in addition to improving the mental health and health-related quality of life (HRQL).

**Objective:**

Investigate the relationship of PAL and their domains with HRQL, mood state (MS) and anxiety. *Method*: 140 Physical Education students (23.6 ± 3.7 years) were evaluated. The Baecke Habitual Physical Activity and Quality of Life (QOL-36) questionnaires, State-Trait Anxiety Inventories (STAI-S and STAI-T) and Profile of Mood States (POMS) scale were used to investigate PAL, HRQL and mental health indicators. Pearson’s correlation coefficient examined the association between PAL and both mental health and HRQL parameters.

**Results:**

There was a correlation between state anxiety and both the domain leisure-time physical activity (LTPA) (p = 0.013) and total PAL score (p = 0.010). In relation to MS, a negative correlation was found between LTPA and total mood disorder (TMD) (p = 0.004). However, there were positive correlations between the vigor subscale and both LTPA (p=0.001) and total PAL (p=0.019). With respect to HRQL, analysis of the relationship between LTPA and total PAL demonstrated positive coefficients with the physical component summary (PCS) (p=0.000; p = 0.005), mental component summary (MCS) (p = 0.000; p = 0.006) and total HRQL (p = 0.000; p = 0.003).

**Conclusion:**

The findings suggest that the rise in LTPA was related to an increase in HRQL and MS. However, PAL was positively related to anxiety.

## INTRODUCTION

Factors associated with technological advances have increasingly distanced individuals from physical activity, given that people are performing fewer tasks, which tend to be facilitated and mediated by technological devices [1]. This problem is one of the causes of a life characterized by lack of movement and exercise (hypokinetic) [1, 2], thereby contributing to the decline in physical activity levels (PAL), which may contribute to the development of chronic degenerative diseases [3], obesity [4] and other disorders [2].

It is interesting to seek or maintain a desirable PAL, since it has a positive impact on the health of individuals [2-4]. To reach the recommended PAL, regular moderate physical activities must be performed, if possible, for 30 minutes a day and 150 minutes a week [5], but leisure-time physical activity (LTPA), which is directly related to HRQL, is also important [6]. In addition to the benefits cited, PAL is an important factor in developing good mental health, correlating primarily with mood state (MS) [7], anxiety [8] and health-related quality of life (HRQL) [9, 10].

The literature corroborates this evidence, showing that regular moderate physical exercise reduces depression and anxiety, in addition to raising mood scores, thereby having a positive effect on mental health [11]. However, if the exercise is intense and inappropriate, especially over a prolonged period of time, all the other variables previously mentioned may worsen, resulting in further problems such as changes in sleep patterns and overtraining [11, 12].

Other studies have also shown the importance of PAL in association with the variables in question, such as the article published by Werneck [7], who obtained positive results related to vigor and humor. In his analysis of 41 adolescents (17 boys and 24 girls), a relationship was observed between physical activity level scores, total mood disorder (TMD) and vigor. This difference showed that the lower the PAL, the higher the TMD, while the highest PAL values exhibited a better correlation with vigor, revealing a beneficial correlation between PAL and mood.

In relation to anxiety, Santos [8] also found a relation with PAL. A group of 200 elderly subjects were divided into two groups of 100, one physically active and the other inactive. It was observed that the inactive individuals obtained higher anxiety scores than their active counterparts. Gordia [9], in turn, found a connection between PAL and HRQL. Of the 608 adolescents studied, the less physically active boys were 1.7 times more likely to exhibit a poor physical domain for HRQL, compared to active boys. The situation was worse for girls, in that the likelihood increased 2.8-fold.

Cross-sectional studies were conducted to determine the relation between PAL and HRQL, showing a consistent association between high HRQL scores and high physical activity levels in apparently healthy adults [13], primarily when LTPA is used [14]. Other authors demonstrated that a high LTPA level was associated with higher scores in the physical component of HRQL in cross-sectional analyses. However, in longitudinal analyses, this increase was related to mental components [15]. Studies also revealed that physical activity affects HRQL more directly through its impact on psychological well-being or emotional function [16].

Despite knowing the harmful consequences that low physical activity levels may have on health, there has been a tendency to decreasing levels in recent years. This phenomenon has been an important field of investigation, underscoring the physical activity levels of young undergraduate students [17], since enrolling into university leads to new social relationships, which, added to academic commitments [18], results in new behaviors, making them vulnerable to circumstances that pose a risk to health [19, 20]. Thus, it is important to investigate these parameters and their associations in this population, in order to guide the planning of actions and health promotion policies, aimed at acquiring and maintaining healthy behaviors [18, 20].

Thus, due to the scarcity of consistent data regarding the association between PAL, mental health and HRQL in Brazil, especially studies with university students, the present study aimed to identify the relation between PAL and anxiety, mood state and quality of life of university students, since they are future examples and disseminators of this information to society.

## METHODS

### Sample

This is a cross-sectional study with a convenience sample composed of 140 physical education students (75 boys and 65 girls) from Veiga de Almeiga University in Rio de Janeiro state (UVA-RJ). Excluded from the sample were undergraduates who: were absent from the class on data collection days; did not complete all the anthropometric assessment tests; refused to participate; did not correctly fill out the questionnaires. Individuals were instructed regarding the study procedures and gave their informed consent in accordance to the 196/96 Resolution of the National Health Council and the Declaration of Helsinki [21, 22]. The project was approved by the Human Research Ethics Committee of the Universidade Veiga de Almeida (UVA - RJ) (no. CAEE 06784012.6.0000.5291) [21].

### Data Collection Procedures

The researchers held a meeting with possible volunteers at the institution. They were explained the goals and ethical procedures and gave their informed consent. Data collection was performed by trained evaluators at two different moments [21]. On the first day the individuals responded to the instruments (STAI, POMS, SF-36 and BAECKE) and on the second day anthropometric variables were collected (Weight and Height). All individuals received the same verbal instructions and any doubts were clarified before the questionnaires were filled out. The instruments also contained written instructions on how to complete them. During the application of the instruments, there was no communication between individuals and there was no time limit imposed [21].

### Anthropometric Variables

The following anthropometric variables were collected: height (cm) using a stadiometer accurate to 0.1 cm (Sanny® ES 2020, Brazil) and weight using a digital scale accurate to 0.01 Kg (Filizola® PL 180, Brazil). Body mass index (BMI) was computed using height and weight measures (weight/height^2^) [21, 23]. All anthropometric measurements were made in accordance with the recommendations of the International Society for the Advancement of Kinanthropometry [24].

### Physical Activity Level

PAL was assessed using the instrument proposed by Baecke **et al.** [25], which consists of 16 questions involving three habitual physical activity scores related to the previous 12 months: (1) daily physical activities carried out in the university and/or at work (occupational physical activity - OPA), consisting of eight questions, (2) sports activities, physical exercise programs and active leisure (leisure-time physical activity - LTPA), composed of four questions, and (3) free time and mobility activities, excluding physical exercises (leisure and locomotion activities - LLA), consisting of four questions. The options are graded on a 5-point Likert scale, except for professional occupation and sport modality [26]. The Ainsworth Compendium of Physical Activities [27] was used to calculate these exceptions in order to determine the metabolic equivalent (MET) of the sport or profession in question. The total score for habitual physical activity and, consequently, the PAL of the subjects assessed, is obtained by adding OPA + LTPA + LLA scores.

### Mood State

Mood state was assessed using the Profile of Mood States Scale [28]. The 42 items on the short form are divided into six subscales: tension (T), depression (D), hostility (H), vigor (V), fatigue (F) and mental confusion (C). Each subscale contains four items rated using a Likert-type scale (not at all - 0; a little - 1; moderately - 2; quite a lot - 3; extremely - 4), with sub-scores ranging from 0 to 16. Subscales T, D, H, F and C are considered negative factors of mood, and V a positive factor. Total mood disturbance (TMD) is calculated by the sum of negative factors, subtracting the positive factor score. The number 100 was added to the final TMD score to avoid negative results [21].

### State-Trait Anxiety

Anxiety was measured by the self-rated STAI scales (State and Trait Anxiety Inventory). The state scale (STAI – S) requires individuals to express how they feel “at the moment” in relation to the 20 items contained on a 4-point Likert scale (not at all - 1; somewhat - 2; moderately so - 3; very much so - 4). The trait scale (STAI – T) also has 20 items, but individuals are instructed to respond how they “generally feel” on a different 4-point Likert scale (almost never - 1; sometimes - 2; often - 3; almost always - 4). The total score of each scale can vary from 20 to 80 points, with scores below 30 indicating a low degree of anxiety, 31-49 a medium level and 50 or more a high degree [21, 29, 30].

### Health-Related Quality of Life

The SF-36 questionnaire (Medical Outcomes Study 36 – Item Short-Form Health Survey) was used to assess quality of life. This instrument contains 36 items, 35 of which encompass eight domains, grouped into two large components: physical (domains: functional capacity, physical aspects, pain and overall health status) and mental (domains: mental health, vitality, social aspects and emotional aspects). A last item assesses change in health over time. Each of these domains received scores between 0 and 100, where zero corresponds to the lowest score and 100 to the highest. Total HRQL was obtained from the weighted mean of the scores of eight domains [21, 31, 32].

### Statistical Analysis

The data were analyzed using the SPSS 20.0 statistical package. The sample was characterized using descriptive statistics, namely central tendency (mean) and dispersion (standard deviation) measures. The Shapiro-Wilk and Levene tests were used to verify sample normality and homogeneity of variances, respectively [21]. Pearson’s correlation was applied to determine the association between PAL and the parameters of mental health and quality of life. A p-value < 0.05 was considered significant.

## RESULTS

A total of 140 undergraduate physical education students, aged 23.6 ± 3.7 years, participated in the study. Mean body weight, height and BMI were 70.7 ± 14.3 kg, 169.4 ± 1.1 cm and 24.5 ± 3.5 kg/m^2^, respectively [21].

Table (**1**) shows the correlation between PAL and the variables HRQL, anxiety and mood state. There was a significant correlation between state anxiety and the LTPA domain and total PAL score. There were no associations between PAL and trait anxiety.

With respect to MS, negative correlations were found between LTPA and the parameters hostility, fatigue and TMD. By contrast, there were positive correlations between the vigor subscale and both LTPA and total PAL.

HRQL showed positive correlation coefficients between LTPA and physical capacity, pain, overall health, vitality, social aspect, emotional aspect, mental health, PCS, MCS and total HRQL. There were also similar correlations between total PAL and physical capacity, pain, vitality, social aspect, mental health, PCS, MCS and total HRQL.

## DISCUSSION

The present study aimed to analyze the correlation between PAL and HRQL, MS and anxiety in physical education students.

In relation to the association between PAL and HRQL, there were no correlations between OPA and LLA. On the other hand, positive correlations were found between the parameters LTPA and PAL and total HRQL total, MCS and PCS. Similar findings were reported in the literature. Brown *et al.* [33], who analyzed 175,850 individuals older than 18 years, reported that physical activity levels are associated with HRQL. Moreover, the authors observed that improvements in HRQL occurred only *via* LTPA, regardless of reaching or not the recommended levels of physical activity aimed at promoting health. Tessier *et al.* [34], in a study with 3,891 individuals (mean age = 51.8 years), showed that a one-hour increase in LTPA per week is associated with a rise in HRQL. Furthermore, Wendel-Vos *et al.* [15] found that a one-hour increase in LTPA per week for 5 years was significantly associated with an improvement in the social aspect of HRQL. In another study that used a relatively large sample of French adults (2333 men and 3321 women), results showed that higher LTPA levels were associated with a higher level of HRQL in both sexes, irrespective of the HRQL domain assessed. According to the same authors, the greater the intensity of LTPA, the higher the HRQL [6]. In a study conducted by Noce *et al.* [35], with 32 healthy university students, the physically active group obtained higher HRQL scores compared to the sedentary group. Brown *et al.* [33], in a study with 175,850 adults enrolled in 2001 in the surveillance system of behavioral risk factors carried out by the U.S. Centers for Disease Control and Prevention (CDC), found that the recommended levels of physical activity are associated with a better total HRQL and self-perceived health status. It was observed that these relations extend across all age groups, both sexes and different ethnicities, and that individuals with higher levels of leisure-time physical activity showed better HRQL.

The relation between LTPA and HRQL can be explained by the enjoyment and distraction normally involved in these exercises, in addition to providing a diversion from heavy work loads that disrupt the daily routine and interferes in mental health [14, 36, 37]. One of the hypotheses that may explain the LTPA-induced improvement in HRQL is related to the pleasure derived from these activities, since the psychological benefits obtained are the result of the mental interpretation of the activity as a pleasant experience [38]. It seems that activities and daily movements in the contemporary world, aspects related to OPA and LLA may trigger stressful situations, which leads us to hypothesize that these parameters have no relationship with perceived HRQV. However, regular physical activity may contribute to reducing stress levels [39].

Thus, PAL was positively related with functional capacity, vitality, emotional aspects and the other HRQL domains, based on the association with vigor and mental health parameters, in addition to improving well-being, satisfaction with body image and self-esteem [40], factors related to leisure-time physical activity [14, 16, 27].

In relation to MS, the research in question revealed negative correlations between LTPA and the parameters hostility (r = -0.188), fatigue (r = -0.213) and TMD (r = -0.243). However, there were positive correlations between vigor (r = 0.285) and both LTPA and total PAL (r = 0.198). Similar associations are reported in the literature, along with the beneficial effects of exercise on mood state [41, 42]. Experimental studies have shown that physical exercise can have a positive impact on the perception of individuals regarding vigor, vitality, physical capacity, overall well-being, satisfaction with physical appearance, self-esteem and sleep quality [11, 43], while evidence also indicates that physical inactivity may be a risk factor for depression [44].

A study involving 17 boys (17.5 ± 1.4 years) and 24 girls (16.0 ± 2.0 years) found a negative relation between PAL and TMD (r = -0.30), and a positive relation between PAL and vigor (r = 0.48), in both girls and boys [7], corroborating the findings of the present investigation. In a 5-month study of 5,341 Norwegian nursing assistants, Eriksen and Bruusgaard [45] observed a relation between LTPA and the risk of developing persistent fatigue. The authors report that nurses who engaged in leisure-time physical activities for 20 minutes or more, at least once a week, displayed less risk of persistent fatigue. Considering the acute effects of physical exercise on MS, studies emphasize that a single session can prompt significant changes, and is a valuable short-term strategy for controlling mood levels in normal individuals and those with disorders [46, 47].

A review study conducted by Werneck *et al.* [38] found that there seems to be positive relation between the individual’s expectation of improved mood and its real change, that is, an inverse relation between pre-exercise mood profile and the psychological benefit achieved. The authors also suggested a possible negative effect on MS after high-intensity exercises, and speculated that an activity at a self-selected intensity may be more advantageous than an imposed one, preferably in a pleasant environment.

A number of hypotheses have been raised to explain the effects of physical activity on MS, namely that the simultaneous interaction of psychological and physiological mechanisms contribute to an improvement in mental health [11, 38]. The hypotheses put forth in a physiological model include a rise in body temperature and cerebral blood flow [48, 49], as well as changes in acyclic monoamines and endorphins [50, 51], which would promote positive psychological effects, decreasing stress and anxiety. According to psychological hypotheses, distraction, social interactions, self-control, self-efficacy, increased self-concept, expectation of change, cognitive assessment or pleasure for the activity would be the factors responsible for improved mood [38].

One conflicting result found in this study was the significant correlation between state anxiety and both LTPA (r = 0.210) and total PAL (r = 0.216). These findings do not corroborate those of Vieira *et al.* [52], who analyzed the effects of two water aerobic sessions per week for 12 weeks in women (n = 16) with mean age of 36.45 (± 9.45) years, diagnosed with anxiety disorder. The authors found a significant reduction in anxiety level in the experimental group. A study with 40 Turkish university students, divided into an experimental and control group, investigated the effects of a 10-week aerobic exercise program on anxiety levels. The results showed a significant decline in trait anxiety scores in those that underwent intervention [53]. Brown *et al.* [54] assessed the implications of aerobic exercise on anxiety levels in patients with disorders and concluded that the results of aerobic exercise were similar to those of other strategies, such as meditation and relaxation, in terms of reducing anxiety, and that they produce better results than their anaerobic counterparts in terms of anxiety [55]. By contrast, predominantly anaerobic exercises should be avoided, since intense exercise is related to symptoms of discomfort, especially due to the possibility of increasing and accumulating blood lactate, a factor that may trigger a panic attack in individuals with anxiety disorder [55, 56].

A number of explanations were suggested to explain the contradictory results found in the analysis between PAL and anxiety. In relation to anxiety levels, young individuals are more vulnerable to changes in behavior caused by inexperience in dealing with the challenges of daily life [17]. As such, anxiety levels tend to decline over time [57]. Another hypothesis may be related to the type of exercise performed, since the instrument used to assess PAL did not differ aerobic from anaerobic exercises, and did not measure other variables related to exercise prescription such as frequency, duration and intensity. Systematic physical exercise programs, particularly aerobic activities [50, 58], in which volume and intensity are commensurate with the needs of the individual, are more effective in producing positive changes in anxiety than occasional programs [57, 59].

Regular aerobic exercise may provide antidepressant and anxiolytic effects [57, 60, 61], and are capable of protecting the organism from the harmful effects of stress on physical and mental health [61]. Physical exercise is an effective strategy in the treatment of anxiety and depression, as are psychotherapy and pharmacological approaches; however, the former are healthier and more economical, in addition to enabling greater adherence than other strategies [50].

Regular physical activity has health benefits and should be encouraged in university settings. Students are constantly faced with assignments, deadlines, long study hours as well as mental and physical exhaustion. It is hoped, therefore, that the results and analyses of the present study contribute to a reassessment of risk behaviors and possible lifestyle changes in university students. The findings may also serve to alert and assist school administrators and professors adopt health promotion strategies aimed at improving mental health and quality of life parameters, not only in the university community, but the population as a whole, since students who are aware of the importance of regular physical activity for their health will also disseminate this information to society.

## LIMITATIONS

The present study has a few limitations, such as the cross-sectional design, precluding establishing a causal relationship. The questionnaires provide an easy, practical and low-cost assessment, but require subjective interpretations of the definitions of intensity, duration and frequency of habitual physical activities, which may occasionally interfere in the quality of information offered for analysis.

## CONCLUSION

PAL showed a positive correlation with vigor and HRQL domains, except for overall health status and physical and emotional aspects. With respect to anxiety, PAL was positively associated with state anxiety. The domains LLA and OPA of PAL showed no relationship with mental health or HRQL. The findings demonstrate a relationship between an increase in PAL and leisure, HRQL and MS. Thus, from practical standpoint, it is suggested that individuals increase their leisure time physical activity, in order to positively affect MS and HRQL.

## Figures and Tables

**Table 1:** Analysis of the relation between physical activity level, anxiety, mood state and health-related quality of life in undergraduate physical education students (Cabo Frio, RJ).

Variables		OPA		LTPA		LLA		PAL	
		*r*	*p*	*r*	*p*	*r*	*p*	*r*	*p *
STAI	State	0.050	0.554	0.210	0.013*	0.129	0.130	0.216	0.010*
	Trait	0.037	0.668	-0.014	0.867	0.040	0.635	0.028	0.740
POMS	Anger	-0.088	0.304	-0.129	0.127	0.073	0.389	-0.095	0.262
	Depression	-0.009	0.914	-0.139	0.101	0.050	0.555	-0.070	0.408
	Hostility	0.000	0.996	-0.188	0.026*	0.054	0.524	-0.095	0.266
	Vigor	-0.042	0.621	0.285	0.001*	0.091	0.285	0.198	0.019*
	Fatigue	0.024	0.781	-0.213	0.012*	-0.059	0.492	-0.148	0.081
	Confusion	0.036	0.673	0.000	1.000	0.054	0.524	0.043	0.612
	TMD	0.000	0.997	-0.243	0.004*	0.012	0.888	-0.148	0.081
HRQL	Total	0.026	0.763	0.317	0.000*	0.082	0.334	0.251	0.003*
	Physical Capacity	0.028	0.739	0.307	0.000*	0.109	0.201	0.257	0.002*
	Physical Aspects	0.030	0.722	0.146	0.084	-0.015	0.859	0.102	0.232
	Pain	0.088	0.300	0.227	0.007*	0.078	0.361	0.225	0.008*
	Overall Health	-0.016	0.851	0.246	0.003*	0.034	0.694	0.162	0.056
	Vitality	-0.025	0.767	0.262	0.002*	0.055	0.521	0.176	0.037*
	Social Aspect	0.020	0.811	0.211	0.012*	0.059	0.487	0.170	0.044*
	Emotional Aspect	0.014	0.873	0.168	0.048*	0.087	0.306	0.152	0.073
	Mental Health	-0.051	0.550	0.342	0.000*	0.084	0.326	0.226	0.007*
	MCS	-0.006	0.945	0.304	0.000*	0.092	0.281	0.230	0.006*
	PCS	0.040	0.640	0.304	0.000*	0.055	0.515	0.238	0.005*

OPA: Occupational Physical Activities; LTPA: Leisure-time Physical Activity; LLA: Leisure and Locomotion Activities; PAL: Physical Activity Level; S-STAI: State Anxiety; T-STAI: Trait Anxiety; POMS: Profile of Mood States Scale; TMD: total mood disturbance; HRQL: Health-related Quality of Life; MCS: Mental Component Summary; PCS: Physical Component Summary; *p < 0.05.
